# Hypothyroidism Alters Uterine Kisspeptin System and Activity Modulators in Cyclic Rats

**DOI:** 10.3390/ijms26020543

**Published:** 2025-01-10

**Authors:** Thayná Queiroz Menezes da Silva, Erikles Macêdo Barbosa, Luciano Cardoso Santos, Luciana Santos de Oliveira, Maria Clara da Silva Galrão Cunha, Isabella Oliveira de Macedo, Brenda Geovana Campos Martins, Cibele Luz Oliveira, Natalia Panhoca Rodrigues, Roberta Araújo-Lopes, Raphael Escorsim Szawka, Juneo Freitas Silva

**Affiliations:** 1 Electron Microscopy Center, Department of Biological Sciences, State University of Santa Cruz, Ilheus 45662-900, Brazil; queirozthayna8@gmail.com (T.Q.M.d.S.); erikles.mb@gmail.com (E.M.B.); lcsantos@uesc.br (L.C.S.); lubaiucha@gmail.com (L.S.d.O.); galrao64@gmail.com (M.C.d.S.G.C.); isabellamacedo1606@gmail.com (I.O.d.M.); bgeovana35@gmail.com (B.G.C.M.); cloliveira.ppgca@uesc.br (C.L.O.); nprodrigues.mev@uesc.br (N.P.R.); 2 Endocrinology and Metabolism Laboratory, Department of Physiology and Biophysics, Federal University of Minas Gerais, Belo Horizonte 31270-901, Brazil; araujo.lopesr@gmail.com (R.A.-L.); reszawka@gmail.com (R.E.S.)

**Keywords:** estrous cycle, kisspeptin, hormone receptors, uterus, thyroid

## Abstract

Hypothyroidism causes ovarian dysfunction and infertility in women and animals and impairs the hypothalamic expression of kisspeptin (Kp). However, kisspeptin is also expressed in the genital system, and the lack of the Kp receptor (Kiss1r) in the uterus is linked to reduced implantation rates. This study investigated the impact of hypothyroidism on the uterine expression of Kp and Kiss1r in female rats throughout the estrous cycle and the associated changes in uterine activity modulators. Hypothyroidism was induced through daily administration of propylthiouracil (PTU) over a period of 14 days. Plasma levels of LH, E_2_, and P_4_, cyclicity, body and uterine weight, uterine histomorphometry, and the gene and/or protein expression of Kiss1, Kiss1r, estrogen receptor α (ERα), progesterone receptor (PR), and thyroid hormone receptor α (TRα) were assessed. Additionally, proliferative activity (CDC-47) and the gene expression of uterine receptivity mediators (*SMO*, *WNT4*, *BMP2*, *HAND2*, *MUC1*, and *LIF*) were evaluated. Hypothyroidism prolonged the diestrus and increased progesterone levels during this phase, while decreasing luteinizing hormone and estradiol on proestrus. In the uterus, hypothyroidism reduced Kp immunostaining on diestrus and *KISS1R* mRNA levels on proestrus. These changes were accompanied by reduced endometrial glands, reduced uterine proliferative activity, and reduced ERα gene and protein expression. Additionally, hypothyroidism led to reduced uterine gene expression of *LIF*, *BMP2*, *WNT4*, and *HAND2*. On the other hand, thyroid hypofunction increased uterine PR and TRα immunostaining, while it reduced *PGR* gene expression on diestrus. These findings demonstrate that hypothyroidism reduces the expression of Kiss1/Kiss1r system in the uterus, which is associated with disrupted uterine estrogen and progesterone signaling and reduced expression of uterine receptivity mediators across the rat estrous cycle.

## 1. Introduction

Thyroid hormones affect uterine function and play a crucial role in normal reproductive processes [1]. Reduction in their plasma levels affects endometrial morphology and embryonic implantation and leads to infertility [2,3]. These changes are due to reduced local signaling through thyroid hormone receptors or alterations in the secretion or expression of other growth factors and hormonal mediators, primarily sex steroids [4,5].

It is well understood that the morphology and physiology of the uterus are primarily regulated by estrogen and progesterone [6]. The cyclical changes in the plasma levels of these hormones regulate the alterations in the uterus throughout the estrous cycle, which are crucial for reproductive success [7,8]. While estrogen promotes endometrial proliferation and influences the expression of mucin 1 and leukemia inhibitory factor (*LIF*), which are crucial during embryonic implantation [6,9], progesterone counteracts cell proliferation triggered by estradiol [8]. Additionally, progesterone facilitates decidualization by regulating genes such as *HAND2*, *BMP2*, *WNT4*, and *IHH*, which are essential for the initiation and maintenance of pregnancy [10,11,12]. However, a recent study revealed that local signaling of kisspeptin, another crucial reproductive peptide encoded by the *Kiss1* gene, also affects uterine physiology via sex steroids. Reduction in uterine expression of the kisspeptin receptor (Kiss1r) in mice led to overexpression of estrogen receptor alpha (ERα) and altered endometrial receptivity, resulting in impaired embryonic implantation, smaller litter sizes, and higher neonatal mortality [13].

Kisspeptin is recognized for its function in controlling the release of gonadotropin-releasing hormone (GnRH) from the hypothalamus [14], which, in turn, influences pubertal development and the menstrual/estrous cycle [15,16]. Reduced hypothalamic expression of kisspeptin was observed in hypothyroid rats [2,17], as well as in the decidua and placenta of these animals [18]. However, it is unclear whether hypothyroidism also impacts the uterine expression of the Kiss1/Kiss1r system throughout the estrous/menstrual cycle.

In women and female mice, kisspeptin shows increased expression in the uterus throughout the secretory/diestrus phase [19,20], a pattern also observed in cats and dogs [21,22]. This suggests a role for kisspeptin in decidualization and embryonic implantation [20]. In fact, reduced kisspeptin signaling in vitro hampers the decidualization of mouse uterine stromal cells [23]. Moreover, knockout mice lacking *KISS1* or its receptor, despite having kisspeptin signaling restored in GnRH neurons, show defects in the development and function of uterine glands [24]. Additionally, they demonstrate reduced uterine expression of *LIF*, a crucial factor for embryonic receptivity [25]. On the other hand, rats with hypothyroidism show reduced endometrial gland development [2], lower uterine expression of *LIF*, and reduced implantation rates [3]. Similarly, hypothyroid women demonstrate reduced decidualization and reduced uterine expression of *HAND2*, *PRL*, and *IGFBP-1* [26]. However, it remains unclear whether hypothyroidism influences uterine responses to sex steroids throughout the reproductive cycle and whether these effects are linked to alterations in the local expression of Kiss1 and Kiss1r.

The hypothesis of this study is that hypothyroidism reduces the expression of the kisspeptin/KissiR system in the uterus of rats throughout the estrous cycle and this alteration is associated with dysregulation of sex steroid hormonal signaling and uterine proliferative activity. Thus, the current study investigated the expression profiles of Kiss1/Kiss1r and the endometrial signaling of estrogen and progesterone in the uterus of hypothyroid rats throughout the estrous cycle.

## 2. Results

### 2.1. Hypothyroidism Affects the Cyclicity and Plasma Levels of LH and Sex Hormones in Rats Throughout the Estrous Cycle

**Figure 1:**
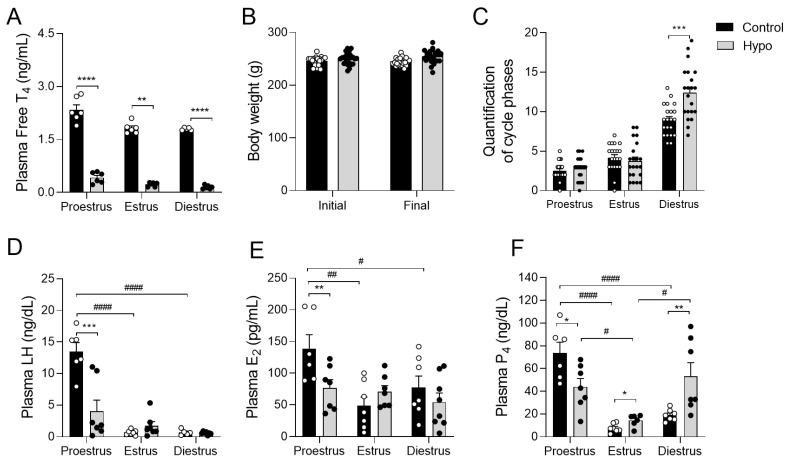
Confirmation of hypothyroidism and evaluation of body weight, reproductive cycles, and hormone levels in hypothyroid rats throughout the estrous cycle. (**A**) Plasma dosage of free T4; (**B**) body weight; (**C**) quantification of the phases of the estrous cycle; (**D**) plasma concentration of LH; (**E**) plasma concentration of estradiol (E_2_); (**F**) plasma concentration of progesterone (P_4_). Significant differences were determined by Student’s *t*-test between groups (*) and ANOVA followed by the Student–Newman–Keuls (SNK) test for different phases of the cycle (#); n= 6–7 animals/group; legends: */# *p* < 0.05; **/## *p* < 0.01; *** *p* < 0.001; ****/#### *p* < 0.0001; LH—luteinizing hormone; P_4_—progesterone; E_2_—estradiol.

The induction of hypothyroidism due to PTU administration was confirmed by a significant reduction in free T4 levels in the hypothyroid animals compared to the controls (Figure 1A). There was no significant difference in body weight between the groups (Figure 1B). Nevertheless, hypothyroidism disrupted the cyclical patterns in the animals, significantly lengthening the diestrous phase compared to the control group (Figure 1C; *** *p* < 0.001). To determine if the altered cyclical patterns seen in hypothyroidism were linked to changes in reproductive hormones, we assessed the plasma levels of LH, E_2_, and P_4_. As expected, for both LH and E_2_, the control animals showed a significant reduction during estrus and diestrus compared to the afternoon of proestrus (Figure 1D,E; #### *p* < 0.0001), confirming the validity of the experimental model used. Hypothyroidism led to a reduction in plasma LH levels during proestrus compared to the control group (Figure 1D; *** *p* < 0.001). A similar reduction was observed in E_2_ levels in the same phase of the cycle (Figure 1E; ** *p* < 0.01). Plasma P_4_ concentrations were lower during estrus in both groups (Figure 1F; # *p* < 0.05; #### *p* < 0.0001). Additionally, there was a significant increase in P_4_ in hypothyroid animals during estrus (Figure 1F; * *p* < 0.05) and a trend toward reduced levels during proestrus (*p* = 0.054) compared to the control group. On the other hand, hypothyroid animals during diestrus showed elevated plasma P_4_ levels compared to the control group (Figure 1F; ** *p* < 0.01).

### 2.2. Hypothyroidism Reduces Uterine Proliferative Activity and Increases Endometrial Expression of TRα in Rats Throughout the Estrous Cycle

**Figure 2:**
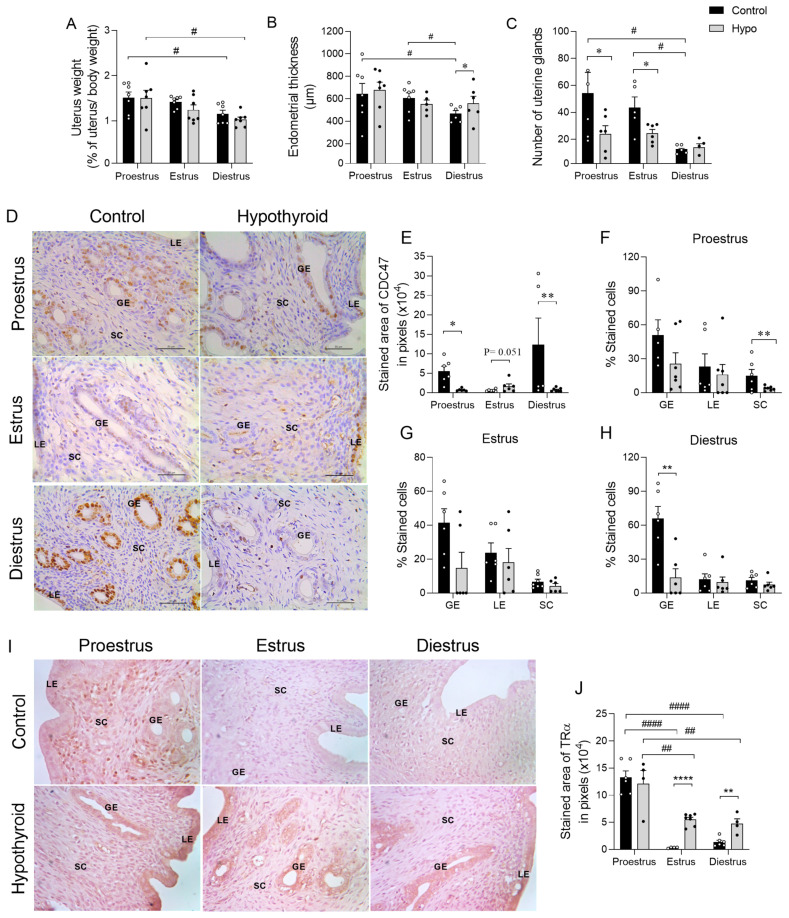
Uterine morphology, proliferative activity, and TRα endometrial immunostaining in hypothyroid rats throughout the estrous cycle. (**A**) Uterine weight; (**B**) endometrial thickness; (**C**) number of endometrial glands. (**D**) Photomicrographs of the immunohistochemical expression of CDC47 in the endometrium (streptavidin-biotin-peroxidase method; counterstained with Harris hematoxylin; scale bar = 50 μm); (**E**) immunolabeling area of the expression of CDC47, measured in pixels. (**F**–**H**) Percentage of cells immunolabeled for CDC47 on proestrus (**F**), estrus (**G**), and diestrus (**H**). (**I**) Photomicrographs of the immunohistochemical expression of TRα in the endometrium (streptavidin-biotin-peroxidase method; counterstained with Harris hematoxylin; scale bar = 50 μm); (**J**) immunolabeling area of the expression of TRα, measured in pixels. Significant differences were determined by Student’s *t*-test between groups (*) and ANOVA followed by the Student–Newman–Keuls (SNK) test for different phases of the cycle (#); n = 6–7 animals per group; legends: */# *p* < 0.05; **/## *p* < 0.01; ****/#### *p* < 0.0001; GE = glandular epithelium; LE = luminal epithelium; SC = stromal cells.

Since hypothyroidism affected the cyclicity and hormonal profile of reproductive hormones, we also examined the uterine morphology of the hypothyroid rats throughout the estrous cycle. In the macroscopic evaluation of the uterus, a significant reduction in weight was observed in both the control and hypothyroid animals in the diestrous phase compared to the proestrous phase (Figure 2A; # *p* < 0.05). However, there were no significant differences in uterine weight between the control and hypothyroid groups across the three phases of the cycle (*p >* 0.05). In the microscopy analysis, control animals showed a reduction in endometrial thickness at diestrus compared to proestrus and estrus (Figure 2B; # *p* < 0.05). Conversely, hypothyroidism was associated with an increased endometrial thickness during diestrus compared to the control group (Figure 2B; * *p* < 0.05). In the quantification of endometrial glands, similar to measurements of endometrial thickness, the control animals showed a reduction in gland number during diestrus compared to proestrus and estrus (Figure 2C; # *p* < 0.05). Hypothyroidism reduced the number of uterine glands at proestrus and estrus compared to the control group (Figure 2C; * *p* < 0.05). Given that hypothyroidism reduced the number of endometrial glands, we also analyzed proliferative activity using CDC47 immunostaining (Figure 2D). There was a notable reduction in the endometrial immunostaining area in hypothyroid animals on proestrus (Figure 2E; * *p* < 0.05) and diestrus (Figure 2E; ** *p* < 0.01) compared to the control group, as well as in the area of myometrial immunostaining in diestrus (Supplementary Figure S1A,B; * *p* < 0.05). Additionally, there was a reduction in the percentage of immunostained stromal cells during proestrus (Figure 2F; ** *p* < 0.01) and of immunostained glandular epithelial cells on diestrus (Figure 2H; ** *p* < 0.01). No significant difference was observed in estrus (Figure 2G). On the other hand, in the evaluation of TRα immunostaining, there was an intense increase in the luminal and glandular epithelium and myometrium in the hypothyroid group compared to the control (Figure 2I), mainly in estrus and diestrus, as confirmed by the analysis of the immunostaining area (Figure 2J; ** *p* < 0.01, **** *p* < 0.0001; Supplementary Figure S1C,D).

### 2.3. Hypothyroidism Disrupts the Uterine Expression of ERα and PR in Rats Throughout the Estrous Cycle

**Figure 3:**
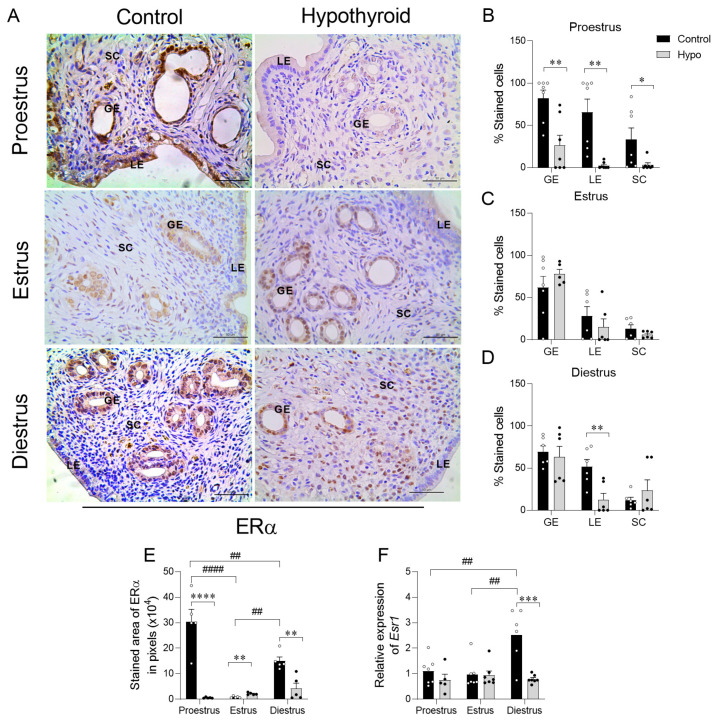
Expression of ERα in the uterus of hypothyroid rats throughout the estrous cycle. (**A**) Photomicrographs of the immunohistochemical expression for Erα in the endometrium (streptavidin-biotin-peroxidase; Harris hematoxylin; scale bar = 50 μm). (**B**–**D**) Percentage of cells immunolabeled for ERα in the luminal and glandular epithelium, as well as in the stroma, on proestrus (**B**), estrus (**C**), and diestrus (**D**). (**E**) Immunostaining area of ERα expression in the endometrium, measured in pixels. (**F**) Relative expression of the *ESR1* gene. Significant differences were determined by Student’s *t*-test between groups (*) and ANOVA followed by the Student–Newman–Keuls (SNK) test for different phases of the cycle (#); n = 5–7 animals per group; legends: * *p* < 0.05; **/## *p* < 0.01; *** *p* < 0.001; ****/#### *p* < 0.0001; GE = glandular epithelium; LE = luminal epithelium; SC = stromal cells.

Since uterine proliferative activity is influenced by sex steroid signaling [7], we assessed endometrial immunostaining and uterine gene expression of ERα and PR. ERα immunostaining was observed in the nucleus and/or cytoplasm across all groups and phases of the estrous cycle and in all endometrial compartments (luminal epithelium, glandular epithelium, and stroma). When comparing immunostaining across different phases of the cycle, control animals showed more pronounced endometrial immunostaining during proestrus compared to estrus and diestrus, with diestrus showing higher staining intensity than estrus (Figure 3E; #### *p* < 0.0001; ## *p* < 0.01). In contrast, hypothyroid animals did not show significant differences in ERα expression across the cycle phases (*p >* 0.05). On proestrus, staining intensity ranged from moderate to high in the control animals, whereas hypothyroidism led to a reduction in the endometrial and myometrial immunostaining area, affecting both nuclear and cytoplasmic levels (Figure 3A,E; **** *p* < 0.0001; Supplementary Figure S1E,F; * *p* < 0.05). In this phase, there was a reduction in the percentage of immunolabeled cells in the luminal and glandular epithelia, as well as in the stroma of hypothyroid animals compared to the control (Figure 3A,B; ** *p* < 0.01; * *p* < 0.05). On diestrus, ERα staining was moderate, predominantly in the endometrial glands. During this phase, hypothyroidism also resulted in a reduction in the endometrial immunostaining area relative to the control (Figure 3E; ** *p* < 0.01), especially in the luminal epithelium (Figure 3D; ** *p* < 0.01). On the other hand, during estrus, despite less intense marking across the endometrium, hypothyroid animals showed an increased area of endometrial and myometrial immunostaining compared to the control group (Figure 3E; ** *p* < 0.01; Supplementary Figure S1E,F; * *p* < 0.05), but there was no significant difference in the percentage of immunolabeled cells on estrus (Figure 3C). In assessing *ESR1* expression, in comparing the different phases of the cycle, control animals showed increased gene expression during diestrus compared to estrus and proestrus (Figure 3F; ## *p* < 0.01). In contrast, no significant differences were observed across the phases in hypothyroid animals. Akin to the immunostaining, we observed a reduction in *ESR1* expression in hypothyroid animals during diestrus compared to controls (Figure 3F; *** *p* < 0.001). However, no significant changes were detected on proestrus or estrus (*p >* 0.05).

**Figure 4:**
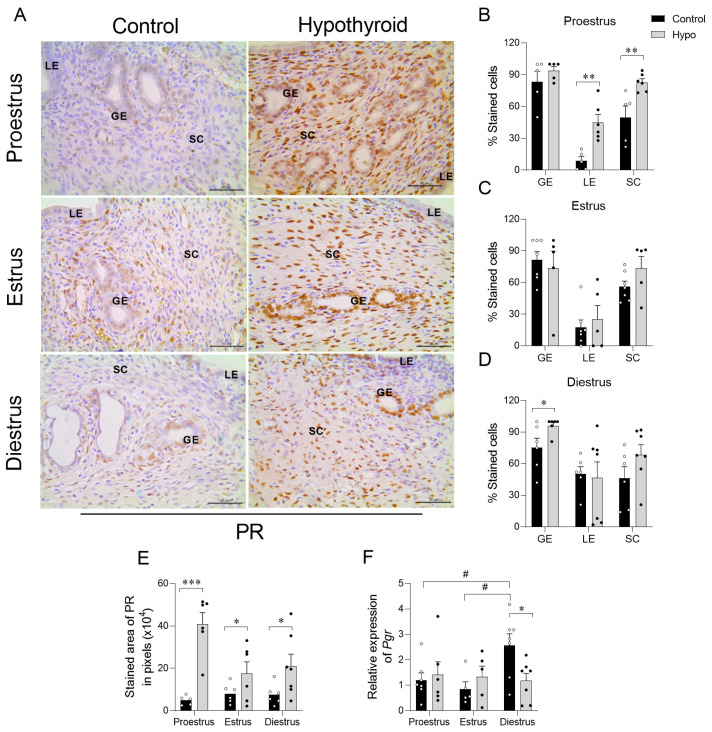
Expression of PR in the uterus of hypothyroid rats throughout the estrous cycle. (**A**) Photomicrographs of PR immunohistochemical expression in the endometrium (streptavidin-biotin-peroxidase method; counterstained with Harris hematoxylin; scale bar = 50 μm). (**B**–**D**) Percentage of cells immunolabeled for PR in the luminal and glandular epithelium and stroma on proestrus (**B**), estrus (**C**), and diestrus (**D**). (**E**) Immunostaining area of PR expression in the endometrium, measured in pixels. (**F**) Relative expression levels of the *PGR* gene. Significant differences were determined by Student’s *t*-test between groups (*) and ANOVA followed by the Student–Newman–Keuls (SNK) test for different phases of the cycle (#); n = 5–7 animals/group; */# *p* < 0.05; ** *p* < 0.01; *** *p* < 0.001; GE = glandular epithelium; LE = luminal epithelium; SC = stromal cells.

Concerning PR, hypothyroidism increased the endometrial and myometrial immunostaining area across all cycle phases compared to the control (Figure 4A,E; *** *p* < 0.001; * *p* < 0.05; Supplementary Figure S1G,H; * *p* < 0.05; *** *p* < 0.001). Additionally, there was a higher percentage of immunostained cells in the luminal epithelium and stromal cells during proestrus (Figure 4B; ** *p* < 0.01) and in the glandular epithelium at diestrus (Figure 4D; * *p* < 0.05). There was no significant difference in the percentage of immunostained cells on estrus (Figure 4C). In the analysis of *PGR* gene expression, similar to that of *ESR1*, a reduction was also observed in the uteri of hypothyroid animals during diestrus compared to the control group (Figure 4F; * *p* < 0.05), and no significant differences were found in other phases (*p >* 0.05). When comparing the different phases of the cycle, no significant differences were observed in the immunostaining area within the groups. However, a significant increase in *PGR* expression was observed in the control animals during diestrus compared to other phases (Figure 4F; # *p* < 0.05). Hypothyroid animals showed no significant differences in *PGR* expression across the different phases of the cycle (*p* > 0.05).

### 2.4. Hypothyroidism Reduces the Uterine Expression of LIF, BMP2, WNT4, and HAND2 Genes in an Estrous-Cycle-Dependent Manner

**Figure 5:**
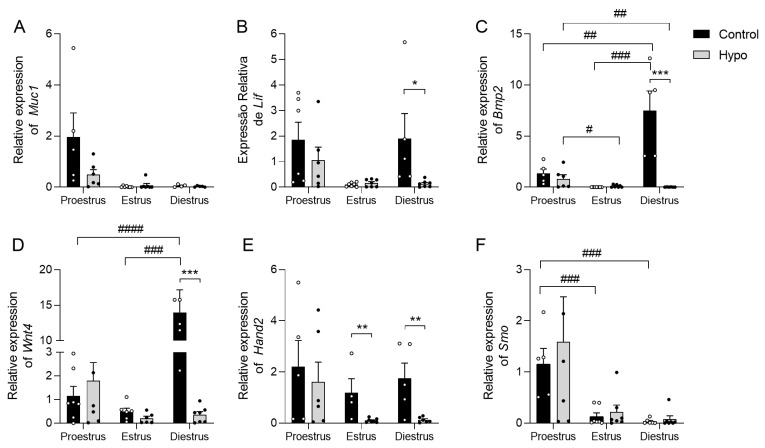
Expression levels of the *MUC1*, *LIF*, *BMP2*, *WNT4*, *HAND2*, and *SMO* genes in the uterus of hypothyroid rats throughout the estrous cycle. (**A**) Relative expression levels of the *MUC1* gene in the uterus. (**B**) Relative expression levels of the *LIF* gene in the uterus. (**C**) Relative expression levels of the *BMP2* gene in the uterus. (**D**) Relative expression levels of the *WNT4* gene in the uterus. (**E**) Relative expression levels of the *HAND2* gene in the uterus. (**F**) Relative expression levels of the *SMO* gene in the uterus. Significant differences between groups were assessed using Student’s *t*-test (*) and ANOVA followed by the Student–Newman–Keuls (SNK) test between the phases of the cycle (#); n = 4–7 animals/group; legends: */# *p* < 0.05; **/## *p* < 0.01; ***/### *p* < 0.001; #### *p* < 0.0001.

Given that hypothyroidism affects the uterine expression of ERα and PR, we aimed to investigate changes in the gene expression of key mediators involved in uterine function that respond to E_2_ and P_4_. To do this, we first assessed the expression levels of *MUC1* and *LIF*, which respond to E_2_ [27,28]. Regarding *MUC1* expression, no significant differences were observed between the groups across the different phases of the estrous cycle (Figure 5A). In contrast, uterine gene expression of *LIF* was reduced in hypothyroid animals during diestrus compared to the control group (Figure 5B; * *p* < 0.05). Regarding the genes responsive to P_4_, there was a significant reduction in the expression of *BMP2* (*** *p* < 0.001), *WNT4* (*** *p* < 0.001), and *HAND2* (** *p* < 0.01) in hypothyroid rats at diestrus compared to the control (Figure 5C–E). A similar reduction in *HAND2* expression was also observed in hypothyroid rats during estrus (** *p* < 0.01). There was no significant difference in *SMO* expression between the groups throughout the estrous cycle (Figure 5F; *p >* 0.05). When comparing the phases of the cycle, significant differences in the expression of *BMP2*, *WNT4*, and *SMO* mRNA were observed in the control group, whereas, in the hypothyroid animals, significant differences were observed only for *BMP2*. Control animals showed higher expression levels of *BMP2* and *WNT4* during diestrus compared to proestrus (## *p* < 0.01; #### *p* < 0.0001) and estrus (### *p* < 0.001) (Figure 5C,D). Conversely, *SMO* expression was elevated at proestrus relative to estrus and diestrus (Figure 5F; ### *p* < 0.001). Unlike the control group, *BMP2* expression in hypothyroid animals was elevated during proestrus compared to estrus and diestrus (Figure 5C; # *p* < 0.05; ## *p* < 0.01).

### 2.5. Hypothyroidism Reduces the Expression of KISS1 and KISS1R in the Uterus in an Estrous-Cycle-Dependent Manner

**Figure 6:**
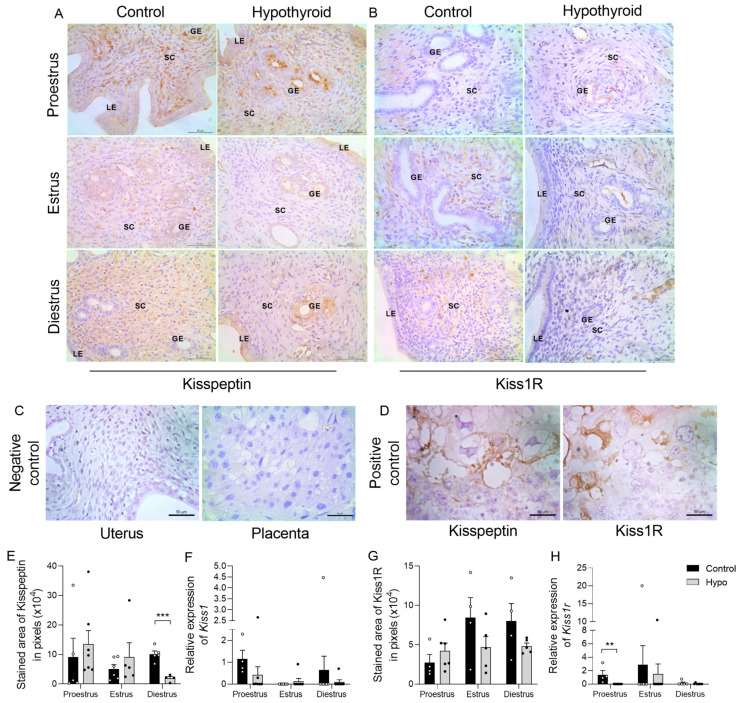
Expression of kisspeptin and Kiss1R in the uterus of hypothyroid rats throughout the estrous cycle. (**A**,**B**) Photomicrographs of the immunohistochemical staining for kisspeptin (**A**) and Kiss1R (**B**) in the endometrium (streptavidin-biotin-peroxidase; Harris hematoxylin; scale bar = 50 μm). (**C**,**D**) Negative control (rat uterus and placenta) (**C**) and positive control (rat placenta) (**D**) (streptavidin-biotin-peroxidase; Harris hematoxylin; scale bar = 50 μm). (**E**,**G**) Immunostaining area of kisspeptin (**E**) and Kiss1R (**G**) in the endometrium, measured in pixels. (**F**,**H**) Relative expression levels of *KISS1* (**F**) and *KISS1R* (**H**). Significant differences between groups were assessed using Student’s *t*-test (*), and differences between the phases of the cycle were evaluated using ANOVA followed by the Student–Newman–Keuls (SNK) test; n = 4–7 animals/group; legends: ** *p* < 0.01; *** *p* < 0.001; GE = glandular epithelium; LE = luminal epithelium; SC = stromal cells.

Since research indicates that kisspeptin plays a role in regulating the expression of ERα in the mouse uterus [13], we also evaluated the protein and gene expression of Kiss1 and its receptor Kiss1R. Kiss1 immunostaining in the uterus was cytoplasmic and ranged from mild to moderate, occurring in all phases of the estrous cycle (Figure 6A). Weak to moderate staining was observed in the luminal and glandular epithelium, primarily during proestrus and estrus, and in the stroma, more prominently in diestrus (Figure 6A). The analysis of the immunostaining area revealed a significant reduction in kisspeptin immunoreactivity in the endometrium of hypothyroid animals during diestrus compared to the control group (Figure 6E; *** *p* < 0.001). No significant differences between groups were observed in other phases of the cycle (*p >* 0.05). The analysis of *KISS1* gene expression revealed no significant differences between the groups across the estrous cycle (Figure 6F). Kiss1R immunostaining, similarly to Kiss1, was cytoplasmic and discrete, observed in the luminal and glandular epithelium as well as in stromal cells, independent of the estrous cycle phase (Figure 6B). The analysis of the immunostaining area revealed no significant differences between the groups (Figure 6G; *p >* 0.05). In assessing the mRNA levels, we observed a significant reduction in *KISS1R* in hypothyroid animals during proestrus compared to controls (Figure 6H; ** *p* < 0.01). No significant differences were observed in the other phases of the estrous cycle (*p >* 0.05).

## 3. Discussion

This study showed that hypothyroidism in rats disrupts the regulation of E_2_ and P_4_ signaling in the uterus throughout the estrous cycle. These alterations may be related to the observed changes in the expression of genes (*LIF*, *BMP2*, *WNT4*, and *HAND2*) that respond to these hormones and are essential for normal uterine function. Additionally, these changes occurred in parallel with reduced expression of Kiss1 and *KISS1R* in the uterus during diestrus and proestrus, respectively. Changes were also observed in cyclicity and the circulating levels of E_2_, P_4_, and LH.

Hypothyroid rats experienced prolonged diestrous phases and showed elevated P_4_ during diestrus, along with reduced LH and E_2_ on the afternoon of proestrus. Normally, there is an increase in LH and E_2_ on the afternoon of proestrus and an elevation in P_4_ during proestrous afternoon and diestrous early morning in rats [29], as observed in the control animals of our study. These findings are consistent with those of De Oliveira et al. [2] and Hatsuta et al. [30], who also observed in hypothyroid rats a prolonged diestrous phase and elevated P_4_ levels, changes attributed to a delayed luteal regression and increased activity of steroidogenic enzymes in the corpus luteum [2,31]. Similarly, Hapon et al. [32] have reported a reduction in E_2_ and LH levels in hypothyroid virgin rats, in line with our results, although they did not report an increase in P_4_. It is established that hypothyroidism hinders the secretion of FSH [30] and also affects the preovulatory surge of LH [33], which impact follicular development and ovulation [2]. These changes may explain the observed reduction in E_2_ levels on proestrous phase and P_4_ levels on the subsequent estrous phase.

Hypothyroidism in rats also reduced the number of endometrial glands during proestrus and estrus, probably due to the reduced plasma levels of E_2_. These findings are consistent with previous research that also observed a reduction in the number of uterine glands in hypothyroid rats during diestrus [2,34]. The smallest number of these glands, observed in the current study on the days of proestrus and estrus, coincided with a reduction in endometrial proliferative activity on proestrus. This was particularly evident in stromal cells and glandular epithelium, as indicated by reduced immunostaining of CDC47 on proestrous and diestrous phases. Accordingly, Kirkland et al. [35] also observed a reduction in the proliferative rate of epithelial, stromal, and muscle cells in the uterus of hypothyroid rats. Furthermore, previous studies involving hypothyroid rats have shown a reduced proliferative rate in other organs, including the placenta [36], corpus luteum [37,38], and ovarian granulosa cells [34].

Of note, the reduction in uterine proliferative activity observed in this study was accompanied not only by lower plasma E_2_ levels but also by reduced uterine ERα expression, along with an increase in uterine TRα and PR immunostaining. It is known that E_2_ stimulates endometrial proliferation through ERα activation [3,6,39]. This receptor also naturally shows increased protein expression on proestrous and diestrous phases [40,41], as observed in the control animals of this study. On the other hand, P_4_ inhibits cell proliferation induced by estradiol [8,42]. Consistent with our findings, Barbanel and Assenmacher [43] reported a reduction in ERα in the uterus, pituitary, and hypothalamus in cases of neonatal hypothyroidism. A previous study conducted in mice indicated that P_4_ treatment throughout the neonatal period inhibits uterine adenogenesis [10]. However, there was a slight increase in ERα immunostaining in the endometrium and myometrium of hypothyroid rats on estrus. This result aligns with observations made by Rodríguez-Castelán et al. [5] in hypothyroid rabbits. But, in contrast to our finding in rats, uterine hyperplasia was found in these animals. However, this increase in ERα immunostaining was of much lower magnitude compared to the receptor suppression in hypothyroid rats on proestrus and diestrus, which has probably determined the overall decrease in the uterine proliferative activity.

Regarding the expression of TRα, which had its endometrial expression increased in hypothyroid animals, similar results were found by Rodríguez-Castelán et al. [5] in evaluating gene transcripts in the uterus of hypothyroid rabbits. It is plausible that this increase in expression is a compensatory reflection of the low circulating thyroid hormone levels presented by these animals because hypothyroid animals showed reduced plasma E_2_ levels and uterine ERα expression and E2 positively regulates uterine TRα expression [44].

Interestingly, the hypothyroid group showed a slight increase in endometrial thickness during diestrus. This result contradicts the observations of Inuwa and Williams [45] and De Oliveira et al. [2], who observed a reduction in endometrial thickness in hypothyroid rats. However, these earlier studies focused on chronic hypothyroidism, induced over periods of six weeks [45] and three months [2] using methimazole and propylthiouracil, respectively. Furthermore, the thickening of the endometrium observed in the current study could be linked to the prolonged diestrous phase and localized edema, which may result from the elevated plasma P_4_ levels observed in these animals [46,47].

Previous studies have shown that ERα expression in the uterus is predominantly nuclear, marking the cells of the luminal and glandular epithelium and stroma [48,49], consistent with our findings. However, during the proestrous and diestrous phases, we observed a significant reduction in cytoplasmic immunostaining of ERα in hypothyroid rats. The cytoplasmic expression of ERα plays a role in activating signaling pathways such as phosphatidylinositol-3-kinase [50,51], indicating that this signaling may be reduced in hypothyroid animals. Indeed, estrogen signaling was reduced in the uterus of these animals, evidenced by reduced expression of the *LIF* gene during diestrus, a gene responsive to E_2_ [28,52] and crucial for implantation [25,53]. Additionally, Shan et al. [3] have shown reduced uterine expression of *LIF* and its receptor at the implantation window in hypothyroid rats. Additionally, mice treated with levonorgestrel [54] demonstrated a reduction in endometrial glands [55] and reduced uterine gene expression of *LIF*, supporting the findings of this study. Hypothyroid rats showed increased plasma P_4_ levels on diestrus and enhanced endometrial and myometrial PR immunostaining. Although the P_4_ increase was restricted to diestrus, a study by Han et al. [56] showed that administering P_4_ in mice elevated PR expression across all endometrial compartments (luminal and glandular epithelium, as well as stromal cells), which supports, at least in part, the increase in uterine PR reported here in hypothyroid rats.

Despite the elevated plasma P_4_ levels and increased uterine protein expression of PR, hypothyroidism led to a reduction in the gene expression of *PGR* on the day of diestrus. This suggests a disruption in uterine signaling by P_4_, potentially due to a downregulation mechanism exerted by P_4_ on its receptor [57]. Although the decreased expression of *PGR* was not reflected in PR immunoreactivity, we found a concomitant reduction in P_4_-responsive genes in hypothyroid animals on diestrus. Hypothyroid rats showed reduced expression of the genes *BMP2*, *WNT4*, and *HAND2*. *BMP2* is essential for decidualization [58] and regulates the expression of *WNT4*, which, in turn, influences cell differentiation throughout the decidual response [59,60]. As indicated by our results, control rats in the diestrous phase show the highest expression of *BMP2* and *WNT4*. The *HAND2* gene, which also showed reduced expression in hypothyroid animals on estrus, inhibits the fibroblast growth factors (FGFs) that are stimulated by estrogen [8]. Supporting our results, Kakita-Kobayashi et al. [26] observed an upregulation of *HAND2* in human uterine stromal cell cultures following T4 treatment.

Given that research in mice has demonstrated that uterine estrogen signaling is influenced by the activity of the Kiss1r [13], and considering that kisspeptin plays a role in embryonic implantation and the decidualization process [13,20,23], we investigated the protein and gene expression of *KISS1* and *KISS1R* in the rat uterus. Interestingly, hypothyroidism led to a reduction in Kiss1 immunostaining in diestrous rats, primarily in the stroma, and also reduced *KISS1R* gene expression during proestrus. A reduced expression of kisspeptin and/or its receptor has also been observed in the hypothalamus [2,17], placenta, and decidua of hypothyroid rats [18]. The reduction in uterine expression of kisspeptin is associated with implantation failures and miscarriage [25,61,62], as well as reduced uterine levels of *LIF* [25], supporting the findings of reduced *LIF* expression in the present study.

Interestingly, Schaefer et al. [13] have shown that conditional knockout mice for *KISS1R* in the uterus display overexpression of ERα. In our study, hypothyroid rats show reduced *KISS1R* gene expression on proestrus, which may have influenced the slight increase in uterine ERα expression during estrus, the day that immediately follows proestrus in the rat estrous cycle [63]. A study on female cats has shown that the expression of the Kiss1r gene and protein in the uterus primarily occurs on the proestrous and estrous phases, mainly in the luminal and glandular epithelium [18]. Furthermore, mice with global ablation of *KISS1R* with rescue of kisspeptin signaling only in GnRH neurons display normal uterine growth but reduced endometrial adenogenesis [24]. These findings are consistent with the reduced endometrial glands observed in the hypothyroid animals in this study.

Our findings show that alterations in the cyclicity and uterine morphology in hypothyroid rats may be linked to disruptions in uterine signaling of E_2_ and P_4_ across the estrous cycle, along with reduced uterine expression of Kiss1 and Kiss1r. These changes possibly contribute to the reproductive dysfunctions associated with hypothyroidism. Understanding these mechanisms provides valuable insights into how hypothyroidism impacts reproductive health, which may guide future research and potential therapeutic approaches to mitigate these effects.

## 4. Materials and Methods

### 4.1. Animals and Experimental Design

Forty-two female Wistar rats (weighing 248 ± 9.9 g) obtained from the Laboratory of Animal Breeding, Care, and Research (LaBIO) at the State University of Santa Cruz (UESC) were used. The animals were housed in plastic boxes under controlled temperature (22 ± 2 °C) and light conditions (12 h light/12 h dark), with free access to water and feed. All experimental procedures were approved by the UESC Animal Use Ethics Committee (Protocol No. 028/22). After showing two complete estrous cycles (proestrus, estrus, and diestrus), the animals were randomly divided into two groups, hypothyroid and control, each consisting of 21 animals. Hypothyroidism was induced by daily orogastric administration of 4 mg/kg of 6-propyl-2-thiouracil (PTU; Sigma-Aldrich, St. Louis, MO, USA) in 3 mL of distilled water [2]. The animals in the control group were administered an equivalent volume of distilled water. The animals were weighed at the start and end of the experiment, and vaginal cytology was conducted daily.

### 4.2. Euthanasia and Sample Collection

Euthanasia was carried out using a guillotine between the 14th and 16th day after initiating treatment with PTU or water, at proestrous (7 animals/group), estrous (7 animals/group), or diestrous 1 (7 animals/group) phases. Blood was collected in heparinized tubes to obtain plasma and then stored at −20 °C for subsequent hormonal dosages. Animals confirmed in diestrus and estrus were euthanized at 10:00 a.m., while those in proestrus were euthanized at 6:00 p.m., coinciding with the luteinizing hormone (LH) peak [64].

In the necropsy, the uterus was collected and fragments from the middle third of each uterine horn were placed in cryotubes containing Trizol, immediately frozen in liquid nitrogen, and stored at −80 °C for gene expression analysis. The remaining uterine horns were fixed in 4% paraformaldehyde for 20 h and then processed using the paraffin embedding technique.

### 4.3. Hormone Level Analysis

Plasma was used to measure the levels of estradiol (E_2_), progesterone (P_4_), LH, and free thyroxine (T4). The Free T4 level was measured using an enzyme immunoassay (ELISA) according to the manufacturer’s instructions (Free T4, IMMULITE, Siemens Medical Solutions Diagnostics, Malvern, PA, USA; Sensitivity: 0.04 µg/dL) [2]. LH levels were measured using an ultrasensitive ELISA, as previously described [65]. Plasma levels of E_2_ and P_4_ were measured using the DRG E_2_ ELISA kit (EIA-2693, DRG Instruments GmbH, Hamburg, Germany) and the rat P_4_ ELISA kit (P4 ELISA, RTC008R, Biovendor Research and Diagnostics Products, Brno, Czech Republic), respectively, according to the manufacturer’s protocols. For each ELISA, all samples were analyzed in a single run.

### 4.4. Histomorphometric Analysis

Uterine sections stained with hematoxylin and eosin (H&E) were imaged using a Spot Color Insight digital camera (SPOT^TM^, Sterling Heights, MI, USA). Four measurements of the endometrial and myometrial thickness were taken at equidistant points using the ImageJ^®^ software version 1.41 (Media Cybernetics Manufacturing, Rockville, MD, USA). The number of endometrial glands present in the entire histological section was also determined [2,34].

### 4.5. Immunohistochemistry

Histological sections of the uterus on silanized charged slides (StarFrost Polycat, Braunschweig, Germany) were subjected to immunohistochemical analysis using anti-PR (1:4000; sc-130071; Santa Cruz Biotechnology, Santa Cruz, CA, USA), anti-ERα (1:500; 6f11; Thermo Fisher Scientific, Waltham, MA, USA), anti-TRα (1:100; 434800, Invitrogen, Carlsbad, CA, USA), anti-KISS-1 (1:100; sc-101246; Santa Cruz Biotechnology, CA, USA), anti-KISS1R (1:200; HPA071913; Sigma-Aldrich, Saint Louis, MO, USA), and anti-MCM2/CDC-47 (1:800; sc-373702; Santa Cruz Biotechnology, CA, USA) antibodies.

The streptavidin-biotin-peroxidase method (Novolink Polymer Detection System, Leica Biosystems Inc., Buffalo Grove, IL, USA) was used. Antigen retrieval was conducted for 20 min using a citric acid solution (0.54 mol/L; pH 6; 98 °C). The slides were incubated in a humid chamber for 42 h with the primary antibody. Incubation stages for blocking endogenous peroxidase and serum blocking were each performed for 30 min. Incubation with the secondary antibody and streptavidin peroxidase was performed for 45 and 30 min, respectively, followed by the application of diaminobenzidine (DAB) (DAB substrate system, Lab Vision Corp., Fremont, CA, USA) as the chromogen. The sections were counterstained using Harris hematoxylin. The negative control was prepared by substituting the primary antibody with phosphate-buffered saline (PBS) and rat placenta was used as a positive control for kisspeptin and Kiss1R (Figure 6C,D) [18].

A detailed and quantitative analysis of the immunohistochemical expression of kisspeptin, Kiss1R, ERα, PR, TRα, and CDC47 in the uterus was conducted. For the quantitative analysis, we used three histological sections per rat. To assess the immunostaining area, images were captured using an Olympus BX-40 microscope (Olympus, Tokyo, Japan) equipped with a Spot Color Insight digital camera (SPOT^TM^, Sterling Heights, MI, USA). Analysis was performed using WCIF ImageJ^®^ software version 1.41 (Media Cybernetics Manufacturing, Rockville, MD, USA). Color deconvolution and thresholding were performed on the images. The data were analyzed and presented as the area of immunolabeling measured in pixels [36]. The assessment of the percentage of immunostained cells for ERα, PR, and CDC47 was conducted by examining 100 cells from the luminal epithelium, 100 cells from the superficial glandular epithelium, 100 cells from the deep glandular epithelium, and 500 cells from the endometrial stroma.

### 4.6. Real-Time qPCR

Total RNA was extracted using Trizol (Invitrogen, Carlsbad, CA, USA). A total of 1 µg of RNA was used for reverse transcription using the GoTaq^®^ qPCR and RT-qPCR Systems kit (A6010, Promega, Madison, WI, USA). The expression levels of the target genes were quantified using SYBR Green-based qPCR on the Applied Biosystems^®^ 7500 Real-Time PCR System. For the reactions, we used 1.5 μL of cDNA, 100 nM of each primer, and 12.5 μL of GoTaq^®^ qPCR Master Mix, 2X, to make a final volume of 20 μL per reaction. As a negative control, the DNA amplification mix was prepared using water instead of the cDNA sample. Additionally, the melting curves of the amplification products were analyzed. The primers for *KISS1*, *KISS1R*, *HAND2*, *WNT4*, *BMP2*, *MUC1*, *LIF*, *ESR1*, *PGR*, and *SMO* were designed based on the mRNA sequences of *Rattus norvegicus* (Table 1). Gene expression was quantified using the 2^−ΔΔCT^ method, with results for each group normalized to the expression of *RPL7* from *Rattus norvegicus* before comparison [66,67].

### 4.7. Statistical Analysis

The data are presented as the mean ± SEM. The unpaired Student’s *t*-test was used to compare groups within each estrous phase, while analysis of variance (ANOVA) followed by the Student–Newman–Keuls (SNK) test was used to compare different phases within each group, using GraphPad Prism 8.0.2^®^ software. The results were deemed significant at *p* < 0.05.

## Figures and Tables

**Table 1:** List of genes and nucleotide sequences for qPCR primers.

Gene	Sequence (5→3)	Product Length	Accession Number
*KISS1R*	F: CAACCTGCTGGCCCTATACC	117	NM_023992.2
	R: TGCAGGGCGCCATCAGT		
*KISS1*	F: GAGCCACTGGCAAAAATGGC	78	NM_181692.1
	R: ATTAACGAGTTCCTGGGGTCC		
*HAND2*	F: GAGGACGGACACGTTACTCG	102	NM_022696.2
	R: TGGGTTCTTGGGCGCTTATT		
*WNT4*	F: TTGTATACGCCATCTCTTCAGCA	84	NM_053402.2
	R: CACAGCCACACTTCTCCAGAT		
*BMP2*	F: TGCTTCTTAGACGGACTGCG	81	NM_017178.2
	R: GGGGAAGCAGCAACACTAGA		
*MUC1*	F: TGTTTCTACCCCTTTCCCGC	100	NM_001398538.1
	R: CTGCGGACTTTTAGGCTTGC		
*LIF*	F: CAGGGATTGTGCCCCTACTG	83	NM_022196.3
	R: GGTGGCATTTACAGGGGTGA		
*ESR1*	F: GCCACTCGATCATTCGAGCA	107	NM_012689.1
	R: CCTGCTGGTTCAAAAGCGTC		
*PGR*	F: CTTCCCAGACTGCACCTACC	76	NM_022847.2
	R: AGGCTGGAATTCGCCGTAAA		
*SMO*	F: CTGACTGGCGGAACTCCAAT	71	NM_012807.2
	R: GCCCACAAAGAAACACGCAT		
*RPL7*	F: TATGTGCCCGCAGAACCAAA	113	NM_001100534.1
	R: TTGAAGATCTGCCGGAGACG		

## Data Availability

The data that support the findings of this study are available from the corresponding author, J.F.S., upon reasonable request.

## Supplementary Materials

The supporting information can be downloaded at: https://www.mdpi.com/article/10.3390/ijms26020543/s1.
