# Supplementary material for: Hypothyroidism Alters Uterine Kisspeptin System and Activity Modulators in Cyclic Rats
Source: Int J Mol Sci. 2025 Jan 10;26(2):543. doi: 10.3390/ijms26020543 (PMC11765193; doi:10.3390/ijms26020543)
Supplement: Supplementary file 1 [file ijms-26-00543-s001.zip › ijms-3281260-supplementary.pdf]

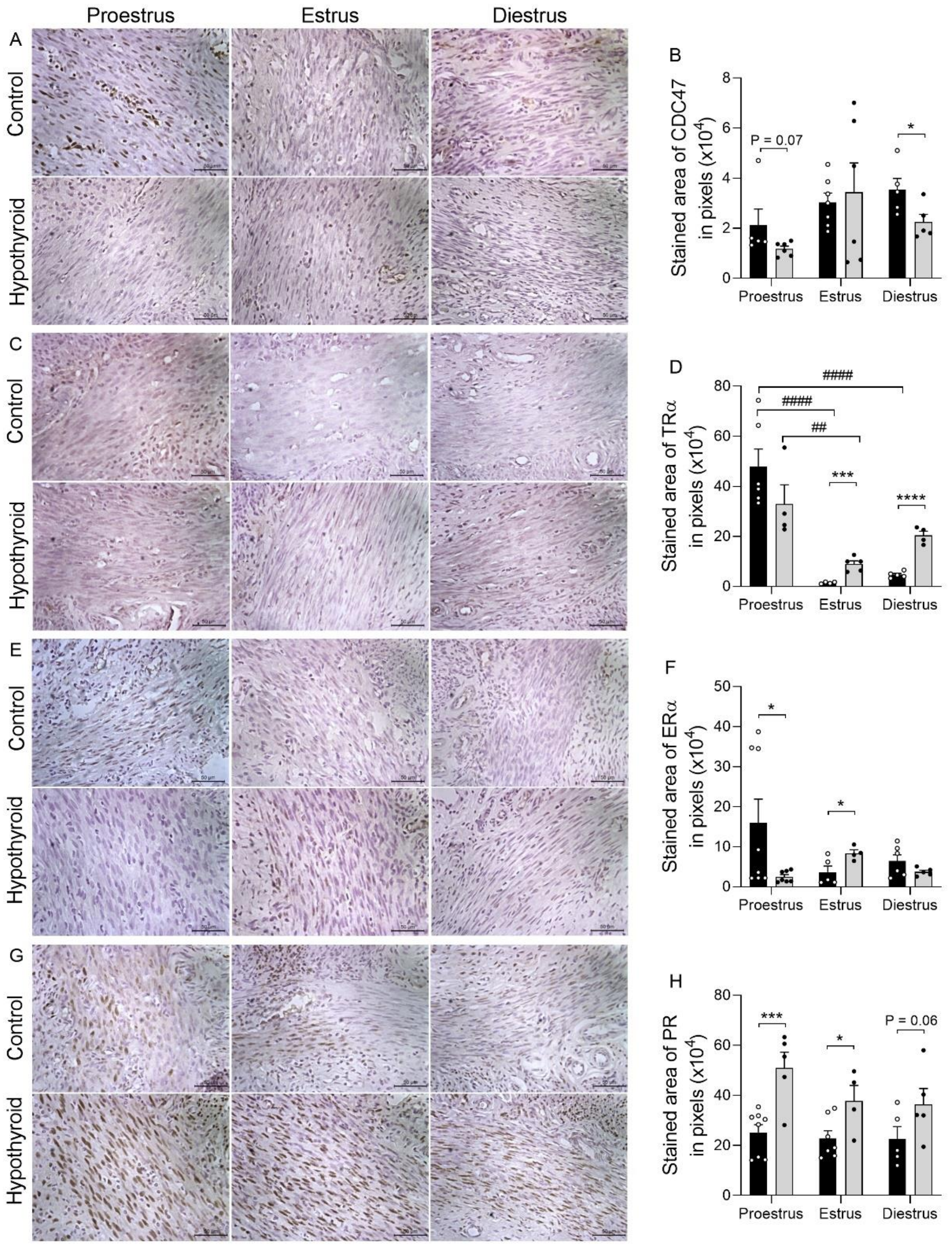

Supplementary figure 1. Myometrial immunostaining of CDC47, TRα, ERα, and PR in hypothyroid rats throughout the estrous cycle. A) Photomicrographs of the immunohistochemical expression of CDC47 (Streptavidin-biotin-peroxidase method; counterstained with Harris Hematoxylin; scale bar = 50 µm); B) Immunolabeling area of the expression of CDC47, measured in pixels. C) Photomicrographs of the immunohistochemical expression of TRα (Streptavidin-biotin-peroxidase method; counterstained with Harris Hematoxylin; scale bar = 50 µm); D) Immunolabeling area of the expression of TRα, measured in pixels. E) Photomicrographs of the immunohistochemical expression of ERα (Streptavidin-biotin-peroxidase method; counterstained with Harris Hematoxylin; scale bar = 50 µm); F) Immunolabeling area of the expression of ERα, measured in pixels. G) Photomicrographs of the immunohistochemical expression of PR (Streptavidin-biotin-peroxidase method; counterstained with Harris Hematoxylin; scale bar = 50 µm); H) Immunolabeling area of the expression of PR, measured in pixels. Significant differences were determined by Student's t-test between groups and ANOVA followed by the Student-Newman-Keuls (SNK) test for different phases of the cycle, with 6-7 animals per group (*P < 0.05, ##P < 0.01, ***P < 0.001, ####/****P < 0.0001). GE= glandular epithelium; LE= luminal epithelium; SC= stromal cells.
